# Estrogens decrease osteoclast number by attenuating mitochondria oxidative phosphorylation and ATP production in early osteoclast precursors

**DOI:** 10.1038/s41598-020-68890-7

**Published:** 2020-07-20

**Authors:** Ha-Neui Kim, Filipa Ponte, Intawat Nookaew, Serra Ucer Ozgurel, Adriana Marques-Carvalho, Srividhya Iyer, Aaron Warren, Nukhet Aykin-Burns, Kimberly Krager, Vilma A. Sardao, Li Han, Rafael de Cabo, Haibo Zhao, Robert L. Jilka, Stavros C. Manolagas, Maria Almeida

**Affiliations:** 1 Division of Endocrinology and Metabolism, Center for Osteoporosis and Metabolic Bone Diseases, University of Arkansas for Medical Sciences, 4301 W. Markham St. #587, Little Rock, 72205-7199 USA; 2 Department of Biomedical Informatics, University of Arkansas for Medical Sciences, Little Rock, USA; 3 Center for Neuroscience and Cell Biology (CNC), University of Coimbra, UC-Biotech, Biocant Park, Cantanhede, Portugal; 4 Department of Orthopedic Surgery, University of Arkansas for Medical Sciences, Little Rock, USA; 5 Division of Radiation Health, Department of Pharmaceutical Sciences, University of Arkansas for Medical Sciences, Little Rock, USA; 6 Central Arkansas Veterans Healthcare System, Little Rock, AR 72205 USA; 7 Translational Gerontology Branch, NIA, NIH, Baltimore, MD USA

**Keywords:** Osteoporosis, Steroid hormones, Apoptosis, Bone

## Abstract

Loss of estrogens at menopause is a major cause of osteoporosis and increased fracture risk. Estrogens protect against bone loss by decreasing osteoclast number through direct actions on cells of the myeloid lineage. Here, we investigated the molecular mechanism of this effect. We report that 17β-estradiol (E_2_) decreased osteoclast number by promoting the apoptosis of early osteoclast progenitors, but not mature osteoclasts. This effect was abrogated in cells lacking Bak/Bax—two pro-apoptotic members of the Bcl-2 family of proteins required for mitochondrial apoptotic death. FasL has been previously implicated in the pro-apoptotic actions of E_2_. However, we show herein that FasL-deficient mice lose bone mass following ovariectomy indistinguishably from FasL-intact controls, indicating that FasL is not a major contributor to the anti-osteoclastogenic actions of estrogens. Instead, using microarray analysis we have elucidated that ERα-mediated estrogen signaling in osteoclast progenitors decreases “oxidative phosphorylation” and the expression of mitochondria complex I genes. Additionally, E_2_ decreased the activity of complex I and oxygen consumption rate. Similar to E_2_, the complex I inhibitor Rotenone decreased osteoclastogenesis by promoting osteoclast progenitor apoptosis via Bak/Bax. These findings demonstrate that estrogens decrease osteoclast number by attenuating respiration, and thereby, promoting mitochondrial apoptotic death of early osteoclast progenitors.

## Introduction

Estrogens protect the adult skeleton from bone loss by slowing the rate of bone remodeling and maintaining a focal balance between bone resorption and formation^1,2^. Estrogen deficiency has the opposite effects. Cell and biochemical studies have strongly suggested that the anti-remodeling effects of estrogens result from their ability to restrain the birth rate of osteoclasts and shorten their lifespan^2–4^. Furthermore, conditional deletion models of the estrogen receptor alpha (ERα)^5,6^, have functionally demonstrated that in females the protective effects of estrogens on trabecular bone are mediated via ERα signaling in cells of the osteoclast lineage^7,8^.

Osteoclasts are highly specialized multinucleated cells responsible for the resorption of the mineralized bone matrix. They arise from mononuclear precursors of the macrophage lineage. Upon stimulation by the macrophage colony-stimulating factor (M-CSF) and the receptor activator of nuclear factor kappa B ligand (RANKL)—the two indispensable stimuli for osteoclastogenesis—the precursors differentiate and fuse to form polykarions^9^. Notably, as the differentiation progresses the number and size of mitochondria and oxidative phosphorylation (OXPHOS) as well as glycolysis and lactate synthesis increase^10–12^.

RANKL prolongs the lifespan of both osteoclast progenitors and mature osteoclasts. Albeit, the survival of osteoclasts is limited by the activation of death pathways that overcome the RANKL-induced pro-survival signals^13^. All osteoclasts die a few days after they are formed, but the identity of the death pathways remains unknown. Activation of Bak and Bax—two pro-apoptotic members of the Bcl-2 family of proteins^14,15^—is indispensable for the permeabilization of the outer mitochondrial membrane and mitochondrial apoptotic cell death. The role of Bak and Bax in apoptosis is redundant and either one of them is sufficient to initiate the apoptosis cascade. Upon an apoptotic trigger Bak and Bax form channels within the outer mitochondrial membrane that permit release of proteins, such as cytochrome c, from mitochondria and consequent activation of the executioner caspases 3 and 7, leading to cell death^16^.

Kato and co-workers proposed earlier that the pro-apoptotic effect of estrogens on osteoclasts is mediated by an increase in FasL production by osteoclasts, causing the activation of the extrinsic death receptor pathway in an autocrine manner^7^. FasL binding to its receptor Fas initiates apoptosis by activating the initiator caspase 8, leading to cleavage and activation of the caspases 3 and 7 and rapid cell death. The extrinsic apoptotic pathway can also stimulate the mitochondrial pathway via caspase 8-mediated cleavage of BID, a pro-apoptotic BH3-only member of the Bcl-2 family, and generation of tBID^17^. The finding that mice lacking FasL or Fas are resistant to OVX-induced bone loss supported a role for Fas/FasL in the anti-osteoporotic actions of estrogens^7,18^. However, several laboratories, including ours, have not been able to confirm a stimulatory effect of E_2_ on FasL production in primary cultures of murine osteoclasts^19–22^. Based on in vitro evidence from an osteoblast-like cell line that estrogen activated ERα signaling directly regulates FasL via an ERE-containing transcriptional enhancer, Krum et al. proposed the alternative idea that FasL stimulates osteoclast apoptosis in a paracrine manner^19^. Yet, work from Wang et al. showing that mice lacking FasL in osteoblast are not protected from the skeletal effects of OVX suggests that osteoblast FasL does not play a major role in the anti-osteoclastogenic actions of estrogens^23^. Furthermore, the idea that estrogens up-regulate FasL in osteoblasts is inconsistent with the fact that estrogens have an anti-apoptotic effect on osteoblasts, even though murine and human osteoblasts express Fas^7,24,25^ and do undergo Fas-mediated apoptosis in response to FasL^26–30^. Hence, heretofore, it remains unclear whether estrogens decrease osteoclast number by promoting the death of osteoclast progenitors, mature osteoclasts, or both. Similarly, the death program involved remains unknown.

In the studies presented herein, we show that estrogens decrease osteoclast number by attenuating mitochondrial respiration and ATP generation and stimulating the Bak and Bax-dependent mitochondrial apoptotic death in early osteoclast precursors, not mature osteoclasts. Furthermore, we show that FasL deficient mice lose bone mass following OVX indistinguishably from FasL-intact control mice, arguing against a role of FasL as a culprit.

## Material and methods

### Animal experimentation

FasL^gld/gld^ mice in the C57BL/6J (B6) background and wild-type B6 mice were purchased from Jackson Laboratories (Bar Harbor, ME). Sixteen week-old FasL^gld/gld^ and wild-type C57BL/6 mice were randomized into sham- or OVX-surgical groups according to their femoral DEXA BMD. BMD measurements were performed 1 day prior to surgery and before euthanasia. Six weeks after surgery animals were sacrificed and the tissues dissected for further analyses. To disrupt the ERα gene in macrophage/monocyte lineage cells ERα^f/f^ mice, generated and maintained in our laboratory^25^, were crossed with mice expressing the Cre recombinase under the control of lysozyme M gene regulatory elements (LysM-Cre)^31^ obtained from Jackson Labs. All mice were in a B6 background. ERα^f/+^ mice heterozygous for the LysM-Cre allele and ERα^f/f^ mice were then intercrossed to obtain ERα^ΔLysM^ and ERα^f/f^ littermate control mice^25^. Bak^+/−^;Bax^+/f^ mice maintained in our facility using breeders donated by Joseph Opferman (St. Jude’s Children’s Research Hospital, Memphis, TN) in a mixed background of 129 and C57BL/6^32^. These mice were interbred to obtain Bak^−/−^; Bax^+/f^ mice, hereafter designated Bak^Δ^Bax^+/f^ mice. Bak^Δ^ mice in the B6 background were obtained from Jackson Labs, and intercrossed with LysM-Cre (B6 background) (25) to obtain Bak^Δ^; LysM-Cre. Bak^Δ^Bax^+/f^ and Bak^ΔLysM-Cre^ mice were then intercrossed to obtain Bak^Δ^; Bax^f/f^; LysM-Cre mice, which are designated Bak^Δ^Bax^ΔLysM^ mice, and Bak^Δ^Bax^f/f^ littermate controls. All mice used in this study were housed under standard laboratory conditions with a 12 h dark, 12 h light cycle and a constant temperature of 20 °C and humidity of 48%. Mice were fed ad-libitum a standard rodent diet (Harlan Teklad 22/5) containing 22% protein, 1.13% calcium, and 0.94% phosphorus.

### Bone density and microarchitecture

Dual energy X-ray absorptiometry (DXA) scans were performed using a PIXImus densitometer (GE Lunar) to quantify bone mineral density (BMD), as previously described^25^. Micro-CT analysis of the distal end of the femora and the vertebrae (L5) was performed after the bones were dissected, cleaned, fixed in 10% Millonig’s formalin and gradually dehydrated into 100% ethanol. Samples were then loaded into 12.3 mm diameter scanning tubes and imaged (model μCT40, Scanco Medical). Scans were integrated into 3-D voxel images (1,024 × 1,024 pixel matrices for each individual planar stack) and a Gaussian filter (sigma = 0.8, support = 1) was used to reduce signal noise. A threshold of 200 was applied to all scans, at medium resolution (E = 55 kVp, I = 145 µA, integration time = 200 ms). The entire vertebral body was scanned with a transverse orientation excluding any bone outside the vertebral body. In the distal femur, 151 transverse slices were taken from the epicondyles and extending toward the proximal end of the femur. The cortical bone and the primary spongiosa were manually excluded from the analysis. All trabecular measurements were made by drawing contours every 10–20 slices and using voxel counting for bone volume per tissue volume and sphere filling distance transformation indices, without pre-assumptions about the bone shape as a rod or plate for trabecular microarchitecture. Cortical thickness was measured at the femoral mid-diaphysis.

### Cell culture

Bone marrow-derived macrophages (BMMs) were obtained, as described previously^33^, from 3 wild-type, ERα^ΔLysM^, Bak^Δ^Bax^ΔLysM^ or respective littermate control mice at 2–3 months of age. Whole bone marrow cells were flushed from femurs and tibias, depleted of red blood cells with ACK buffer, and plated in α-MEM phenol red free complete media (10% fetal bovine serum, 100 U ml^−1^ penicillin, and 100 µg ml^−1^ streptomycin) with macrophage colony-stimulating factor (M-CSF; 10 ng/ml; R&D Systems Minneapolis, MN). Twenty-four hours later, non-adherent cells were re-plated in Petri dishes with M-CSF (30 ng/ml) for 4 days to obtain BMMs, which were used as osteoclast precursors. To generate pre-osteoclasts and mature osteoclasts, BMMs were cultured in α-MEM complete media with M-CSF (30 ng/ml) and receptor activator of nuclear factor kappa B ligand (RANKL; 30 ng/ml; R&D Systems Minneapolis, MN) for 2 and 4 days, respectively, with or without E_2_ (10^−8^ M, Sigma-Aldrich, St. Louis, MO). Osteoclasts were fixed with 10% neutral buffered formalin for 10 min and stained for tartrate-resistant acid phosphatase (TRAP), using the Leukocyte Acid Phosphatase Assay Kit, following the manufacturer’s instructions (Sigma-Aldrich, St. Louis, MO). A pre-osteoclast was defined as a round mononuclear TRAP-positive cell and an osteoclast as a multinucleated (> 3 nuclei) TRAP-positive cell. Cells were plated, at least, in triplicate for all TRAP staining assays. To investigate the effects of Rotenone on apoptosis of osteoclast precursors, Rotenone (5 nM) or vehicle (DMSO) was added for the first 24 h of the culture period.

### Seahorse mitochondrial flux analysis

The BMMs were plated and treated with RANKL (30 ng/ml) for 48 h with or without E_2_ (10^−8^ M). The media in the wells was replaced with XF assay media and the plate was kept in a non-CO_2_ incubator for 20 min at 37 °C. After recording three total cellular respiration measurements with the XF96 analyzer, oligomycin (10 µg/ml) was added to inhibit mitochondrial ATP synthase and measure the decrease in oxygen-consumption rate that is linked to ATP turnover. FCCP (10 µM, an uncoupler) was used to determine the maximal respiration potential of the cells. The amount of non-mitochondrial oxygen consumption was determined by inhibiting the respiratory chain activity with an antimycin A and rotenone cocktail (10 µM). These data were used to calculate the mitochondrial basal respiration, ATP-linked respiration, reserve respiratory capacity, and proton leak as we previously described^34,35^.

### ATP production

ATP levels were measured by a luciferin-luciferase based assay using an ENLITEN ATP assay system bioluminescence detection kit (Promega, Madison, WI) according to the manufacturer’s protocol as previously described^25^. In brief, BMMs were plated on 12-well plates with M-CSF (30 ng/ml) and RANKL (30 ng/ml) in α-MEM complete media with or without E_2_ (10^−8^ M). After 24 h, cells were washed with PBS and extracted with 100 µl of 0.5% trichloroacetic acid (TCA) in ATP-free water. Each extracted sample (10 µl) was neutralized by adding a Tris-acetate buffer (90 µl) adjusted to pH 7.75. The luciferase reagent (25 µl) was added immediately into the neutralized samples (10 µl) before measurement using a luminometer. A standard curve for intracellular ATP was obtained with a series of dilution of the ATP standards and ATP-free water provided in the kit. ATP was normalized for protein concentration.

### Quantitative RT-PCR

Total RNA was extracted from cultured cells using TRIzol reagent (Invitrogen, Carlsbad, CA) and reverse-transcribed using the High-Capacity cDNA Archive Kit (Applied Biosystems, Grand Island, NY) according to the manufacturer’s instructions. Taqman quantitative RT-PCR was performed as previously described^36^. The primers and probes for murine ERα (Mm00433148_mH), Tfam (Mm00447485_m1), PGC1-β (Mm00504720_m1), Cathepsin K (Mm00484039_m1), Bak (Mm00432045_m1) and Bax (Mm00432050_m1) were manufactured by the TaqMan® Gene Expression Assays service (Applied Biosystems). Relative mRNA expression levels were normalized to the house-keeping gene ribosomal protein S2 (Mm00475528_m1) using the ΔCt method^37^. To quantify mitochondrial DNA, total DNA was extracted from cultured cells using QIAamp DNA Mini Kit (Qiagen, Valencia, CA) the following Taqman assays for the mitochondrial genes ND2 (Mm04225288_s1) and CytB (Mm04225271_g1) were quantified by Taqman quantitative RT-PCR. Relative DNA expression levels were calculated using the Tfrc copy number reference assays (Applied Biosystems) and the ΔCt method, as above.

### Microarray analysis

Cells were harvested for RNA isolation as described above. One µg total RNA per sample was hybridized to MouseRef-8 v1 Expression beadchips (Illumina, San Diego, CA) following protocols listed on the Gene Expression and Genomics Unit of the NIA (https://www.grc.nia.nih.gov/branches/rrb/dna/index/protocols.htm). Microarray florescent signals were extracted using an Illumina BeadArray 500GX reader. The data analyses of microarray were performed in R software. The signals on each sample are preprocess and normalized using lumi package^38^. The microarray data of this study is deposited at GEO database under accession number GSE111237 (https://www.ncbi.nlm.nih.gov/geo/query/acc.cgi?acc=GSE111237). The intrinsic variability of individual microarray sample was firstly evaluated using principle component analysis. The differential gene expression analysis between ERα^f/f^ and ERα^ΔLysM^ group at different cell types, which are BMMs, pre-osteoclasts and mature osteoclasts, was identified by moderate *t* test through limma package^39^. The calculated *p* values were adjusted for multiple testing using Benjamini Hochberg method. The adjusted *p* value were used for gene set enrichment analysis of Gene Ontology (GO) using PIANO package^40^. The selected GO terms that have enrichment *p* value lesser than 10E^−5^ were chosen and plot as a heatmap.

### Apoptosis assays

Caspase-3 activity was measured by determining the degradation of the fluorometric substrate DEVD-AFC (Biomol Research Labs, Plymouth, PA) and protein concentration was measured using a Bio-Rad detergent–compatible kit (Bio-Rad, Hercules, CA), as described previously^33,41^. In brief, BMMs were plated on 96-black well plates with M-CSF (30 ng/ml) and RANKL (30 ng/ml) in α-MEM complete media with or without E2 (10^−8^ M). After 24 h, the cultured cells were lysed the cells in 20 mM Tris-HCl (pH 7.5), 150 mM NaCl, 1 mM EDTA, 10 mM NaF, 1 mM sodium orthovanadate, 5 mg ml^−1^ leupeptin, 0.14 U ml^−1^ aprotinin, 1 mM phenylmethylsulfonylfluoride, and 1% Triton X-100. Cell lysates were incubated with 50 mM DEVD-AFC in 50 mM HEPES (pH 7.4), 100 mM NaCl, 0.1% CHAPS, 10 mM DTT, 1 mM EDTA, and 10% glycerol. The released fluorescent signal was measured in a microplate fluorescence reader with excitation/emission wavelengths of 340/542 nm.

### Western blot analysis

BMMs, pre-osteoclasts and mature osteoclasts were washed twice with ice-cold PBS and lysed with a buffer containing 20 mM Tris-HCL, 150 mM NaCl, 1% Triton X-100, protease inhibitor mixture, and phosphatase inhibitor cocktail (Sigma-Aldrich)^25^. After incubation on ice for 30 min, the cell lysates were centrifuged at 13,200 rpm for 15 min at 4 °C. Protein concentration of cell lysates were determined using the DC Protein Assay kit (Bio-Rad). The extracted protein (40 µg per sample) was subjected to 8–10% SDS-PAGE gels and transferred electrophoretically onto polyvinyl difluoride membranes. The membranes were blocked in 5% fat-free milk/Tris-buffered saline for 90 min and incubated with each primary antibody followed by secondary antibodies conjugated with horseradish peroxidase. The following monoclonal antibodies were used: ERα (Santa Cruz Biotechnology, Santa Cruz, CA; sc-8002, 1:500), Lamin B (Santa Cruz Biotechnology, sc-373918, 1:500), p-IκB (Cell Signaling, Denver, MA; #9,246, 1:1,000), NFATc1 (Santa Cruz Biotechnology, sc-7294, 1:5,000), and β-actin (Santa Cruz Biotechnology, sc-81178, 1:2,000). We also used rabbit polyclonal antibodies for IκB (Santa Cruz Biotechnology, sc-847, 1:500), RelB (Cell Signaling, #4,954, 1:1,000), and p65 (Abcam, Cambridge, MA; ab7970, 1:1,000).

### Nuclear fraction

Cells were washed twice with ice-cold PBS, lysed with cytosolic extraction buffer (50 mM Tris-HCl (pH 8.0), 2 mM EDTA, 0.5% Nonidet P-40, 20% glycerol, and 0.5 mM phenylmethylsulfonyl fluoride) for 5 min on ice, and then micro-centrifuged at 4,000 rpm for 5 min. Supernatants were used as cytosolic extracts and the pellet was lysed in nuclear extraction buffer (20 mM HEPES (pH 7.6), 420 mM NaCl, 2 mM EDTA, 1% Triton X-100, 20% glycerol, 25 mM β-glycerophosphate, and 0.5 mM phenylmethylsulfonyl fluoride) for 30 min on ice and was then micro-centrifuged at 12,000 rpm for 15 min, as previously described^42^. Nuclear extracts were used for Western blot analysis as described above.

### Isolation of mitochondria

BMMs cultured to 80–100% confluence in a T175 flask were used to isolate mitochondria. A cell pellet was collected and resuspended in PBS and Digitonin (4 mg/mL) (Sigma-Aldrich), and incubated on ice for 10 min. After addition of 1 ml of PBS the homogenate was centrifuged for 10 min at 10,000 g. The supernatant was removed and the pellet was resuspended in PBS and centrifuged. This process was repeated twice. Mouse heart mitochondria were used as positive control. Heart tissue obtained from a C57BL/6 mouse was homogenized and mitochondria were isolated using a differential centrifugation procedure^43^ in a buffer containing 75 mM sucrose, 225 mM mannitol, 50 mM Tris-HCl and 35 mM BSA at 4 °C. The homogenate was centrifuged at 740 g for 3 min at 4 °C. The pellet was discarded and the supernatant centrifuged for 5 min at 740 g and for 10 min at 10,000 g. The mitochondrial pellets from both BMMs and heart were washed and resuspended to a final protein concentration of 3.5 mg/mL.

### Gradient blue native gel electrophoresis (BN-PAGE)

A BN-PAGE system was assembled with gradient Mini-PROTEAN® TGX™ Precast Gels (4–15%) (BioRad). The inner compartment was filled with cathode buffer containing 50 mM Tricine (Sigma-Aldrich), 15 mM Bis Tris and 0.01% Brilliant Blue G. Twenty µL of each sample (containing 70 µg of protein) were loaded into each well. The outer compartment was filled with anode buffer containing 50 mM Bis Tris and the gel was run at 75 V. When the blue front reached one-third of the gel the cathode buffer was replaced with one devoid of brilliant blue G. Gel was then run at 150 V until the blue front reached the bottom of the gel, in the tank surrounded by ice. After removal from the cast the gel was incubated at 37 °C with 50 mL Complex I substrate, containing 1 M Tris-HCl, 4 mg of NADH and 10 mg of nitrotetrazolium blue chloride (in 200 µL EtOH), for 10 min with shaking. Gel images were developed using a VersaDocTM imaging system (Bio-Rad).

### Statistics

All in vitro assays were repeated at least once. Group mean values were compared, as appropriate, by Student’s two-tailed *t* test or one-way ANOVA or two-way ANOVA with Tukey’s test, after determining that the data were normally distributed and exhibited equivalent variances.

### Statement

All experiments and methods were performed in accordance with relevant guidelines and regulations. All animal procedures were approved by Institutional Animal Care and Use Committees of the University of Arkansas for Medical Sciences and the Central Arkansas Veterans Healthcare System, Protocol # AUP 3,684.

## Results

### Estrogens decrease osteoclast numbers by acting on early osteoclast progenitors, not on mature osteoclasts

**Figure 1:**
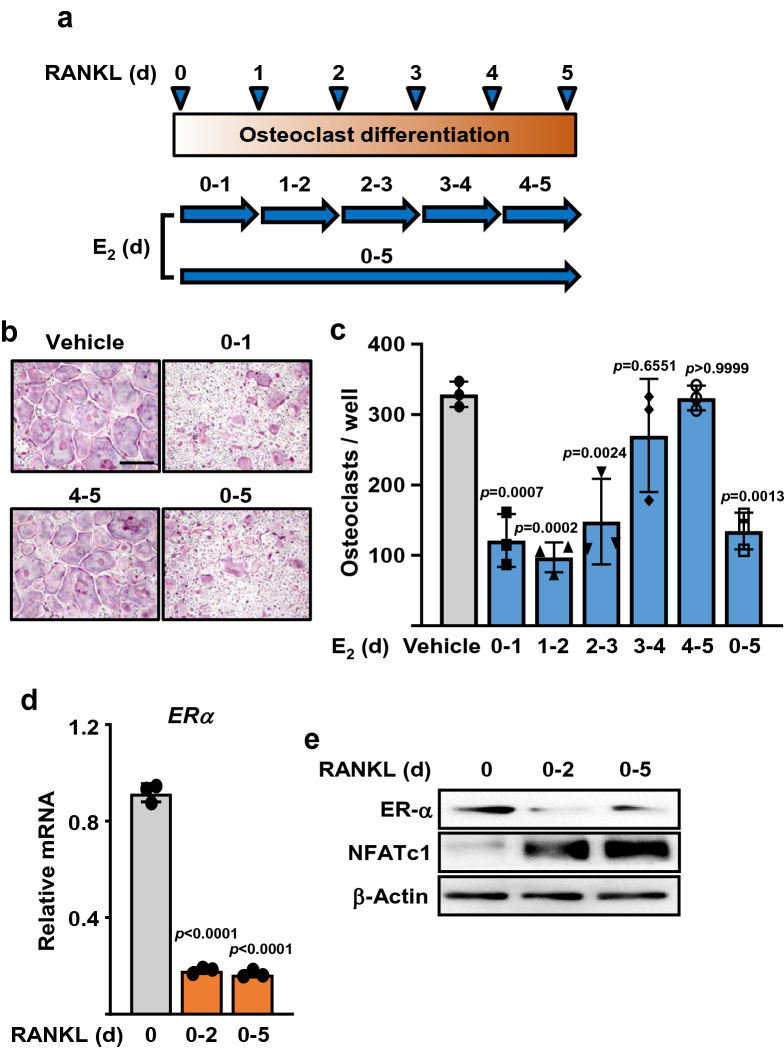
Early osteoclast progenitors are major targets of the antiosteoclastogenic actions of E_2_. (**a**) Time course used to identify the E_2_-sensitive stages during osteoclast differentiation. (**b**) Representative pictures and (**c**) number of TRAP-positive multinucleated osteoclasts derived from BMMs cultured with M-CSF, RANKL, and E_2_ following the time course described in (**a**). Scale bar, 500 µm. (**d**) ERα mRNA and (**e**) protein levels in BMMs treated with M-CSF and without or with RANKL for 2 or 5 days to obtain pre-osteoclast and mature osteoclast, respectively. Data represent mean ± S.D.; *p* values versus untreated cells analyzed by 1-way ANOVA.

To determine the stage of osteoclast differentiation at which estrogens act to decrease osteoclast number we cultured BMMs from wild-type C57BL/6 mice for 5 days in the presence of M-CSF and RANKL. E_2_ at a concentration of 10^−8^ M was added into the cultures at the beginning of day 1, 2, 3, 4, or 5, and removed 24 h later (by changing the medium) (Fig. 1a). In a parallel set of cultures, we added E_2_ at the beginning of day 1 and kept it throughout the 5-day period. The presence of E_2_ during only the first 24 h decreased the number osteoclasts at the end of the 5-day culture as much as it did when E_2_ was present throughout the entire 5-day period (Fig. 1b,c). Addition of E_2_ after day 3, a time when mature osteoclasts had already begun to form, had no effect on the number of osteoclasts at the end of the 5 days (Fig. 1b,c). These observations clearly indicate that during the RANKL-induced differentiation process estrogens act on very early osteoclast progenitors, not on mature osteoclasts.

Interestingly, the mRNA levels of ERα were highest in BMMs not exposed to RANKL (i.e. time 0 of the culture) and dramatically decreased thereafter in cultures treated with RANKL for 2 or 5 days (Fig. 1d). Similarly, ERα protein levels declined as the levels of the osteoclast specific transcription factor NFATc1 increased (Fig. 1e). A decrease in ERα expression with osteoclast differentiation was also noted earlier in human cells and in the monocytic line RAW264.7^44–46^. Nonetheless, estrogen was effective in decreasing osteoclasts in day 3 of culture, when ERα levels seem to be already lower. Along with evidence that estrogens promote the apoptosis of mature osteoclasts^2–4^, our findings suggest that differences in receptor levels are not responsible for the early effects of estrogens during osteoclastogenesis.

### Estrogens promote osteoclast progenitor apoptosis via Bak and Bax

**Figure 2:**
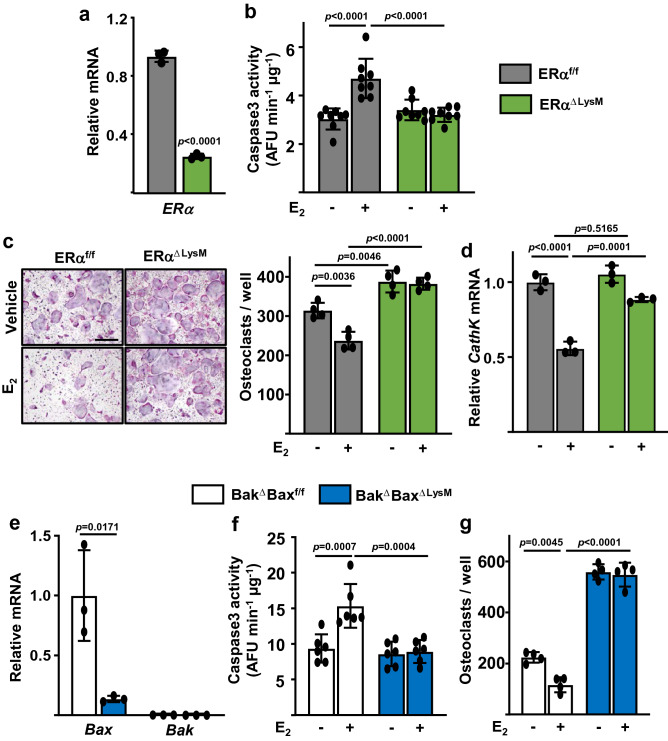
E_2_ inhibits replication and stimulates apoptosis of early osteoclast progenitors in a Bak/Bax-dependent fashion. BMMs of the indicated genotypes were cultured with M-CSF and RANKL in the presence or absence of E_2_. (**a**) mRNA levels of ERα in BMMs cultured with M-CSF for 24 h. (**b**) Apoptosis determined by caspase-3 activity in cultures maintained for 24 h. (**c**) *left*, Representative pictures and *right*, number of TRAP-positive multinucleated osteoclasts in cultures maintained for 5 days, E_2_ or vehicle was added for only the first 24 h of the culture period. Scale bar, 500 µm. (**d**) mRNA levels of Cathepsin K in cultures described in (**c**). (**e**) mRNA levels of Bax and Bak in BMMs cultured with M-CSF for 24 h. (**f**) Apoptosis measured using the same conditions as in (**g**). (**f**) Number of TRAP-positive multinucleated osteoclasts quantified using the same conditions as in (**c**). Data represent mean ± S.D.; *p* values analyzed by two tail unpaired *t* test (**a**, **e**) or 2-way ANOVA (**b**–**d**, **f**, **g**).

Based on the results of the experiment described in Fig. 1, we proceeded to investigate the mechanism of action of estrogens in osteoclast progenitors. In these experiments, we first used BMMs from mice with conditional deletion of ERα in cells of the myeloid lineage expressing LysM-Cre and littermate controls with intact ERα^25^ (Fig. 2a). BMMs cultures from either genotype were maintained for 24 h in the presence of both RANKL and 10^−8^ M E_2_. E_2_ stimulated caspase 3 activity in cells with intact ERα^f/f^ but had no effect in ERα-deficient cells (Fig. 2b). Consistent with this result, E_2_ decreased RANKL-induced osteoclast formation in ERα-intact, but not ERα-deficient, cells (Fig. 2c). Similar findings were obtained when we quantified the mRNA expression of Cathepsin K, a marker of mature osteoclast (Fig. 2d).

To probe into specific death mechanisms, we next used BMMs from mice with global deletion of Bak and deletion of Bax in cells of the myeloid lineage (Bak^−/−^; Bax^f/f^; LysM-Cre mice), referred to hereafter as Bak^Δ^Bax^ΔLysM^ mice (Fig. 2e). In cultures of BMMs from control mice lacking only Bak (Bak^Δ^Bax^f/f^), E_2_ stimulated caspase-3 activity, as measured at the end of the first day of culture (Fig. 2f). The effects of E_2_ on caspase-3 activity were abrogated in cells from Bak^Δ^Bax^ΔLysM^ mice. The number of osteoclasts determined after 5 days of culture was much higher in the cultures lacking Bak and Bax (Fig. 2g), most probably because osteoclast lifespan was prolonged in these cultures. Lack of both Bak and Bax also prevented the reduction in osteoclast number caused by E_2_ treatment during the first 24 h of the 5-day cultures (Fig. 2g).

Collectively, these findings suggest that the decrease of osteoclast number by estrogens is due, at least in part, to activation of the mitochondrial apoptotic death pathway in osteoclast progenitors rather than mature cells.

### FasL ligand plays no role in ovariectomy (OVX)-induced loss of bone in mice

**Figure 3:**
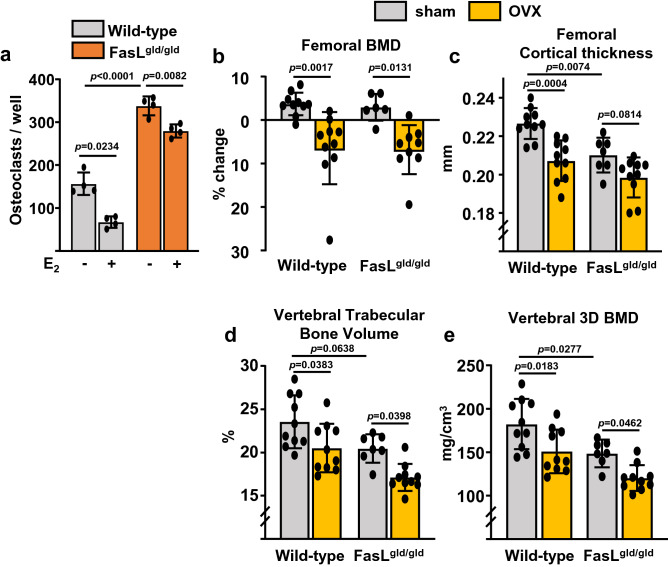
Loss of bone mass with OVX is unaffected by FasL deletion. (**a**) Number of TRAP-positive multinucleated osteoclasts in cultures maintained for 5 days and treated with E_2_ or vehicle for only the first 24 h of the culture period. (**b**–**e**) Wild-type and FasL^gld/gld^ mice were sham-operated or ovariectomized for 6 weeks. (**b**) Percent change in BMD, determined by DXA 1 day before surgery and before sacrifice. (**c**) Cortical thickness in femur midshaft, and (**d**) trabecular bone volume per tissue volume and (**e**) cancellous BMD (3D BMD) in L5 by micro-CT. n = 7–10/group. Data represent mean ± S.D.; *p* values analyzed by 2-way ANOVA.

It has been been proposed earlier that an increase in FasL is responsible for the pro-apoptotic actions of estrogens on osteoclasts^7,19^. To determine whether FasL is indeed involved in the direct effects of estrogens on osteoclast progenitors, we used cells from mice lacking FasL. Bone marrow-derived cultures from FasL^gld/gld^ formed higher number of osteoclasts than cultures from wild type mice (Fig. 3a), perhaps reflecting a higher number of pre-osteoclasts in FasL^gld/gld^ mice. This result is in line with earlier findings that FasL^gld/gld^ mice have increased osteoclast numbers^47^. E_2_ treatment during the first 24 h of the 5-day cultures decreased osteoclast formation in both wild-type and FasL^gld/gld^ cultures (Fig. 3a). To elucidate the contribution of FasL to the protective effects of estrogens on the skeleton in the in vivo setting, we next ovariectomized mice lacking FasL. In these experiments FasL^gld/gld^ mice in C57BL/6 background were randomized into two groups and were sham-operated or ovariectomized at 16 weeks of age. Wild type C57BL/6 mice of the same age served as controls. Bone mineral density (BMD) by dual energy X-ray absorptiometry (DXA) was measured 1 day before surgery and 6 weeks later, immediately prior to sacrifice. The loss of uterine weight upon OVX (determined after the animals were sacrifice at 22 weeks of age) was indistinguishable between the two genotypes (data not shown). After 6 weeks of estrogen deficiency, wild-type control mice showed the expected decrease in BMD (Fig. 3b). A similar decrease was observed in FasL^gld/gld^ mice (Fig. 3b). Because the effects of estrogen on trabecular and cortical bone compartments are mediated via different mechanism and DXA BMD cannot distinguish these compartments, we proceeded and performed micro-computerized tomography (CT) analysis of vertebrae and femurs from sham-operated and OVX FasL^gld/gld^ and control mice. As shown before^8,48,49^, OVX decreased both cortical bone thickness and trabecular bone volume and BMD (Fig. 3b–e) in wild-type controls, determined at the femoral mid-shaft and L5, respectively. Sham-operated FasL^gld/gld^ mice had decreased cortical and trabecular bone mass when compared to sham-operated wild-type mice. Nevertheless, and in contrast to the previous reports, the loss of bone mass in FasL^gld/gld^ mice with OVX was indistinguishable from the control mice (Fig. 3b–e). Together with the in vitro data, these findings suggest that FasL does not play a major role in the protective effects of estrogens on the skeleton.

### E2 inhibits mitochondrial genes in osteoclast progenitors

**Figure 4:**
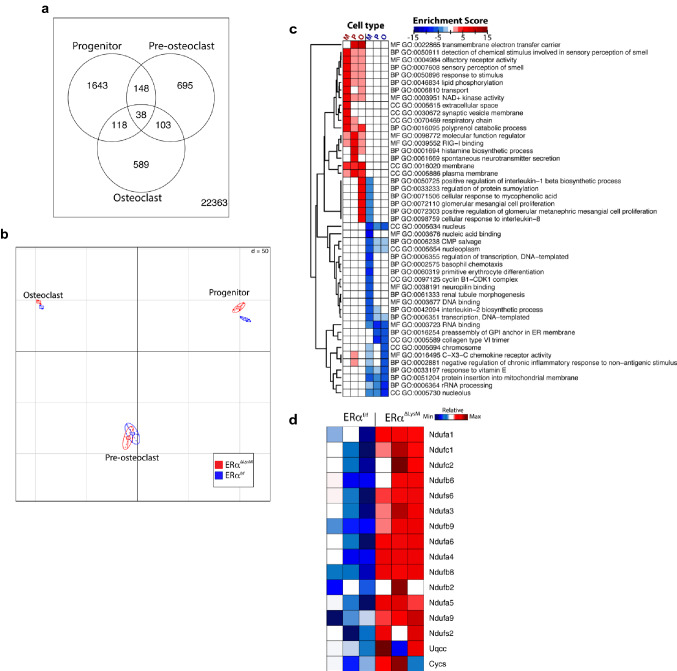
Gene expression profiling in osteoclastic cells lacking ERα. (**a**) A Venn diagram shows overlap between ERα-regulated genes in osteoclast progenitors, pre-osteoclasts and mature osteoclasts. (**b**) Principle component analysis shows intrinsic variability of transcriptome among different samples in the study. (**c**) Functional enrichment heat map based on Gene Ontology of genes up-regulated or down-regulated by ERα deletion in LysM-Cre targeted cells. M = BMMs, P = pre-osteoclasts and O = mature osteoclasts. (**d**) Gene expression heat map of mitochondria complexes I–V genes that have an adjusted-*p* value < 0.05.

To investigate the molecular mechanism(s) responsible for the pro-apoptotic effect of estrogens on osteoclasts, we went on to perform an unbiased transcriptomic analysis in cells from ERα^ΔLysM^ mice and ERα^f/f^ littermate controls. In these experiments, BMMs were generated by culturing whole BM cells with M-CSF alone for 5 days, and pre-osteoclasts and osteoclasts by culturing the BMMs with both M-CSF and RANKL for 2 and 4 days, respectively (19). Microarray analysis was performed in cells treated for 4 h with 10^−8^ M E_2_. Principal-component and hierarchical clustering analysis strongly supported the distinct nature of each population. It also indicated that most genes significantly affected by ERα were in macrophages and pre-osteoclast, not in mature osteoclasts (Fig. 4a,b). Differential expression analysis indicated that the pre-osteoclast and osteoclast population expressed high levels of osteoclast markers, like for example Ctsk, calcitonin receptor, and TRAP (data not shown), confirming the differentiation stage of the three populations. We next performed gene-set enrichment analysis using Gene Ontology and PIANO (Platform for Integrated Analysis of Omics data)^40^ to identify cellular processes affected by estrogens. ERα-deleted osteoclast progenitors and mature osteoclasts exhibited significant enrichment for “transmembrane electron transfer carrier”, suggesting that ERα signaling inhibits oxidative phosphorylation (Fig. 4c).

Because of our finding that the primary targets of estrogen action are osteoclast progenitors, we focused our attention on the genes altered by estrogen in this cell population. We then used gene set enrichment analysis (GSEA)^50^ to broadly test for enrichment of well-defined gene sets from the comprehensive Molecular Signature Data Base (MSigDB), version 5.1 (www.broadinstitute.org) and Ingenuity Pathway Analysis (IPA) for RANKL-dependent genes, which are downregulated by estrogen in ERα positive versus ERα negative cells. The expression of 14 of the 46 genes that encode mitochondrial complex I proteins^51^ were higher in ERα-deleted BMM. They include Ndufa1, 3, 4, 5, 6, 9, Ndufb2, 6, 8, 9, and Ndufc1, 2, Ndufs2, 6 (Fig. 4d). Expression of Uqcc (complex III), and Cycs, the electron carrier between complex III and IV, was also increased.

### E2 attenuates oxidative phosphorylation and ATP production in osteoclast precursors

**Figure 5:**
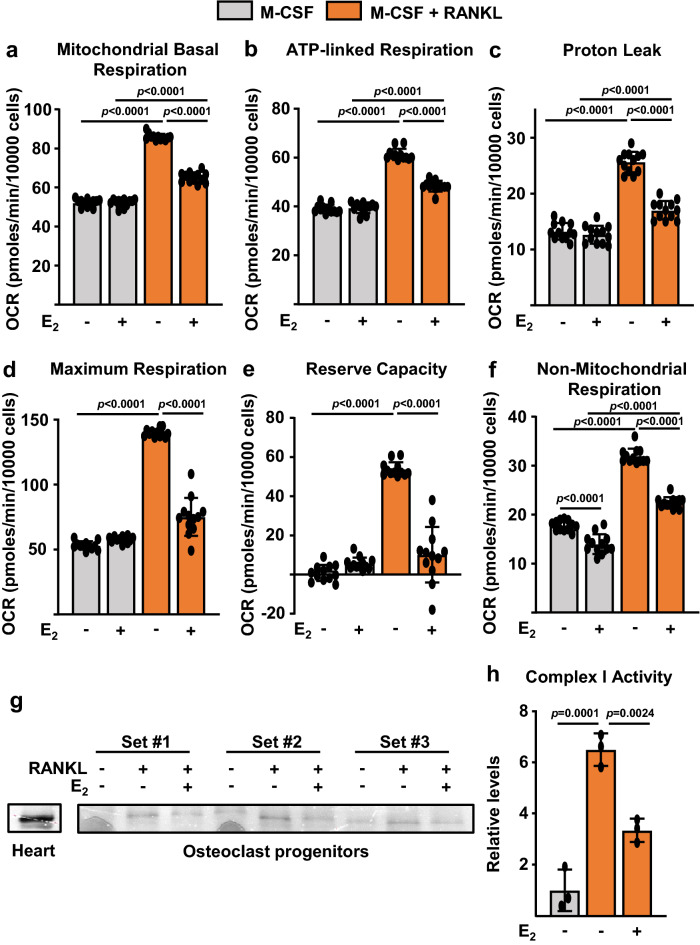
E_2_ attenuates mitochondria and non-mitochondrial respiration in osteoclast progenitors. BMMs were cultured with M-CSF, or with M-CSF and RANKL for 2 days. (**a**–**f**) Different fractions of mitochondrial and non-mitochondrial respirations per cell, in cells untreated or treated with E_2_ for 48 h, measured by Seahorse. n = 12–14/group. Data represent mean ± S.D.; *p* values analyzed by 2-way ANOVA. (**g**–**h**) BMMs were cultured with M-CSF and RANKL in the presence or absence of E_2_ for 6 h. (**g**) Complex I activity using non-gradient blue native gel electrophoresis of mitochondria isolated from BMMs cultured with M-CSF and RANKL in the presence or absence of E_2_ for 6 h. (**h**) The activity of complex I was quantified based on the average intensity of bands from three samples per treatment normalized for protein content. Data represent mean ± S.D.; *p* values analyzed by 2-way ANOVA (**a**–**f**) or 1-way ANOVA (**h**).

**Figure 6:**
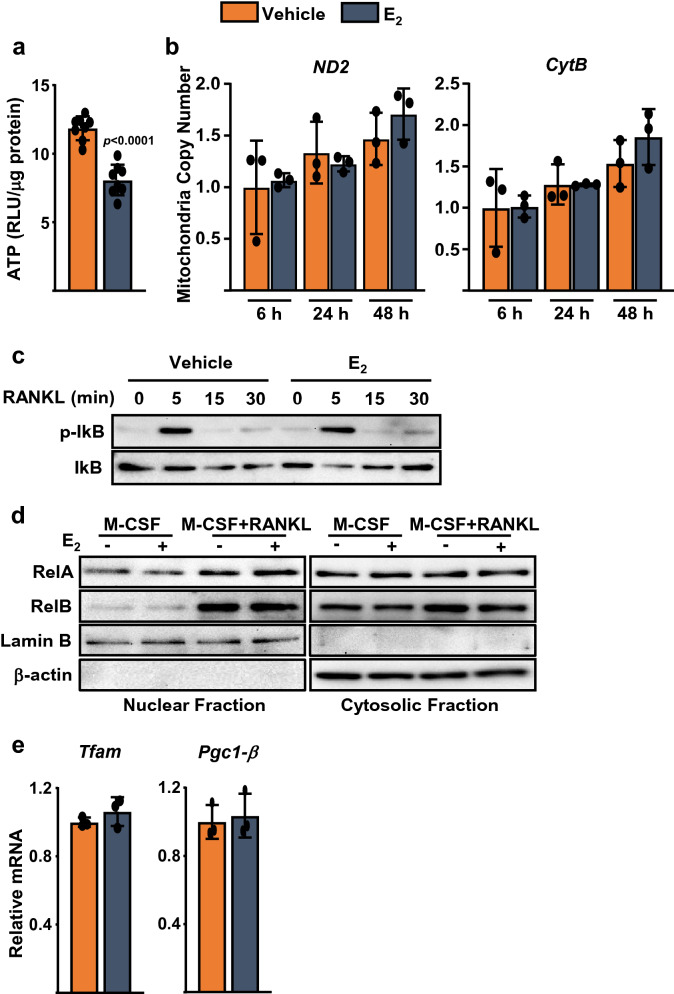
E_2_ attenuates ATP production in osteoclast progenitors. (**a**) ATP levels in BMMs treated with M-CSF and RANKL for 24 h in the presence or absence of E_2_; RLU, relative luminescence units, Data represent mean ± S.D.; *p* values analyzed by two tail unpaired *t* test. (**b**) Ratio of mitochondrial:nuclear DNA determined in BMMs treated with M-CSF and RANKL with or without E_2_ for the indicated times. (**c**, **d**) Protein levels by Western blotting in the whole cell lysates, nuclear, or cytosolic fractions of BMMs pre-treated with E_2_ for 30 min, followed by RANKL for the indicated time points (**c**), or for 6 h (**d**). (**e**) mRNA levels in BMMs treated with M-CSF and RANKL with or without E_2_ for 24 h.

Prompted by the results of the microarray, we went on to examined whether estrogens alter oxidative phosphorylation, by performing extracellular flux analysis in osteoclast progenitors. In BMMs from wild-type C57BL/6 mice, stimulation with RANKL for 48 h increased mitochondrial basal respiration, ATP-linked respiration, proton leak, maximum respiration (maximal electron transport chain activity), reserve respiratory capacity [the difference between the maximum oxygen consumption rate (OCR) and basal respiratory OCR, that provides cells flexibility during increased energy demands], and non-mitochondrial respiration (Fig. 5a–f)^52^. E_2_ had no effect in most of these mitochondrial measurements in cells not treated with RANKL (Fig. 5a–e), but effectively prevented the increase in mitochondria respiration caused by RANKL in most of these measurements. In addition, E_2_ inhibited non-mitochondrial respiration in both the absence and presence of RANKL (Fig. 5f). We next examined whether E2 could inhibit complex I activity using gradient blue native gel electrophoresis (BN-PAGE). BN-PAGE was developed for the separation of mitochondrial membrane proteins and complexes in the mass range of 10 KDa–10 MDa^53^. In‐gel activity of Complex I was assessed after separation of the protein complexes and incubation with the electron donor NADH, the substrate for NADH dehydrogenase. Six hours of exposure of BMMs to RANKL increased complex I activity, and this effect was greatly attenuated by E_2_ (Fig. 5g,h). Cardiac mitochondria isolated from mouse heart were used as positive control (Fig. 5g). In line with the inhibitory actions of E_2_ on respiration, E_2_ attenuated RANKL-stimulated ATP production (Fig. 6a).

It is well established that RANKL increases mitochondria biogenesis during osteoclastogenesis^10,12^. To examine whether E_2_ could alter mitochondrial content, we measured the DNA levels of two genes encoded by the mitochondria genome ND2 and Cytochrome b (normalized to genomic DNA content). E_2_ had no effect on the levels of the two genes up to 48 h, suggesting that the inhibitory actions of E_2_ on complex I activity and respiration are independent of changes in mitochondrial content (Fig. 6b). RANKL stimulates mitochondria biogenesis, at least in part, via the non-canonical NF-kB pathway and RelB^54^. Therefore, we examined whether E_2_ altered RANKL-induced canonical or non-canonical NF-kB activity. As expected, RANKL stimulated the phosphorylation of IkB and the nuclear accumulation of RelA or RelB, respectively (Fig. 6c,d). E_2_, however, had no impact on the NF-kB pathway in the presence or absence of RANKL. Likewise, E_2_ had no effect on the levels of PGC1-β and Tfam, two transcription factors that regulate mitochondrial biogenesis (Fig. 6e).

### Rotenone mimics the effects of E_2_ on osteoclast progenitors

**Figure 7:**
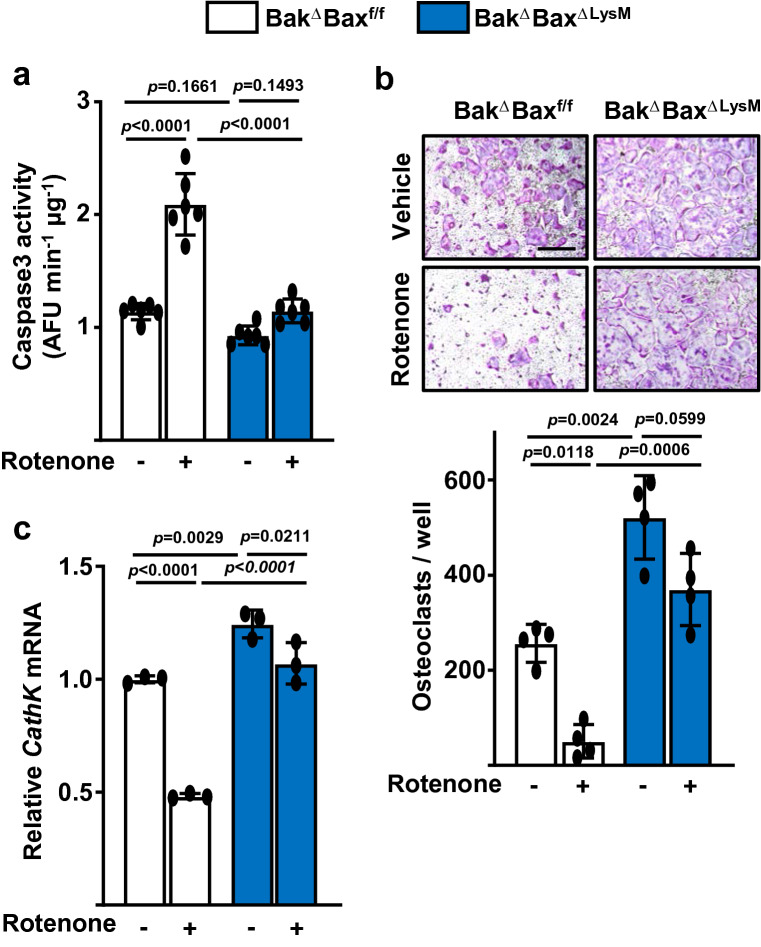
Rotenone decreases osteoclastogenesis by stimulating apoptosis of early osteoclast progenitors. (**a**) Apoptosis in BMMs treated with M-CSF and RANKL for 24 h in the presence or absence of Rotenone. (**b**) *top*, Representative pictures and *bottom*, number of TRAP-positive multinucleated osteoclasts derived from BMMs cultured with M-CSF and RANKL for 5.5 days. Rotenone or vehicle (DMSO) was added for the first 24 h of the culture period. Scale bar, 500 µm. (**c**) mRNA levels of Cathepsin K in cultures described in (**b**). Data represent mean ± S.D.; *p* values analyzed by 2-way ANOVA.

Finally, to independently ascertain whether inhibition of complex I alone is sufficient to promote osteoclast progenitor apoptosis and a decrease in osteoclastogenesis, we examined the effects of rotenone, a specific inhibitor of mitochondrial complex I. Similar to E_2_, treatment with rotenone for 24 h increased caspase 3 activity in osteoclast progenitors from control mice (Fig. 7a). The pro-apoptotic effect of rotenone was abrogated in Bak and Bax-deficient cells from Bak^Δ^Bax^ΔLysM^ mice. Likewise, the presence of rotenone during only the first 24 h decreased osteoclastogenesis, as determined by enumeration of osteoclasts (Fig. 7b) and expression of Cathepsin K (Fig. 7c), at the end of the 5-day culture. The suppressive effect of rotenone on osteoclastogenesis was attenuated in cells lacking Bak and Bax (Fig. 7b), indicating that the pro-apoptotic effect contributes to the inhibitory actions of this agent.

## Discussion

The anti-resorptive effects of estrogens in trabecular bone are mediated via ERα signaling in cells of the myeloid lineage leading to a decrease in osteoclast number. However, heretofore the downstream mediators have remained unclear. We and others had previously shown that E_2_ stimulates mature osteoclast apoptosis. Our findings in the present report indicate that these pro-apoptotic actions on mature osteoclasts do not contribute significantly to the inhibitory effects of E_2_ on osteoclastogenesis. Instead, our results suggest that estrogens promote the apoptosis of osteoclast progenitors and these effects, most likely, account for the decrease in osteoclastogenesis. Early inhibitory effects of estrogens during osteoclast differentiation were also reported in human cells and in murine RAW264.7 cells^44–46^. Most cell death in vertebrates occur via the intrinsic or mitochondrial pathway of apoptosis which requires Bak and Bax^55^. Both proteins are necessary for apoptosis in response to a diverse array of intrinsic death signals e.g. ER stress, DNA damage and a rise in intracellular ROS, and extrinsic death receptor signals. We found here that estrogens cause apoptosis of osteoclast progenitors and decrease osteoclast formation via Bak and Bax. We have previously elucidated that the mitochondrial death pathway decreases osteoblast number and bone mass^56^.

Earlier work had implicated the pro-apoptotic Fas/FasL system as a mediator of the inhibitory actions of estrogens on bone resorption^7^. FasL can promote apoptosis via Bax/Bak-dependent or independent mechanisms. We show here that E_2_ decreases osteoclastogenesis in osteoclast progenitors lacking FasL, but not in cells lacking Bak/Bax suggesting that Bak/Bax stimulation, independently of FasL, is responsible for the decrease in osteoclast formation. These evidence together with the findings that FasL^gld/gld^ mice lose bone mass following OVX indistinguishable from controls does not support the contention that FasL plays a critical role in the effects of estrogens on bone^19–22^. The reasons for the different results between our study and that of Nakamura et al. with the same FasL^gld/gld^ mice are unclear. One possible explanation for this discrepancy is the fact that in the previous work mice were estrogen deficient for just 2 weeks, in contrast to 6 weeks in our study. In addition, unlike Nakamura et al., we used microCT to quantify bone mass. Defects in the Fas/FasL system cause severe immune system disorders, which have been well characterized in Fas (Lpr) or FasL (Gld) deficient mice^57–59^. It is plausible that an altered immune response due to different environmental conditions also contributes to the discrepant results.

It has been previously shown that mitochondria biogenesis is stimulated as early as 48 h after addition of RANKL^10,54,60^. We show here that RANKL increases complex I activity as early as 6 h, well before an effect on mitochondria content can be detected. These findings suggest that RANKL exerts stimulatory effects on mitochondrial respiration that precede changes on mitochondria biogenesis. RANKL promotes mitochondria biogenesis, at least in part, via upregulation of the PPARγ coactivator Ppargc1β^10^. NF-kB signaling via RelB and NIK is also required for mitochondrial biogenesis in response to RANKL, and this requirement is independent of Ppargc1β^54^. However, we found no effect of E_2_ on mitochondrial content, Ppargc1β levels or the alternative NF-kB signaling. In contrast, E_2_ inhibited the early stimulatory effects of RANKL on complex I activity. This action of E_2_ is associated with decreased expression of genes involved in oxidative phosphorylation. Specifically, using an unbiased approach, we found that ERα signaling in osteoclast progenitors attenuates the expression of several genes of the mitochondrial complex I. The mammalian NADH dehydrogenase (complex I)—the largest respiratory chain complex—oxidizes NADH produced by the tricarboxylic acid cycle and the β-oxidation of fatty acids, reduces ubiquinone in the inner mitochondrial membrane, and supplies the rest of the electron transport chain with electrons to reduce O_2_ to water. It is also a major source of reactive oxygen species^61^. Natural estrogens such as 17α-estradiol, E_2_, and estrone, or the synthetic estrogen diethylstilbestrol (DES) inhibit mitochondrial electron transport in rat uterus, liver, and skeletal muscle^62,63^. Additionally, studies in isolated liver mitochondria indicate that E_2_ leads to mitochondrial failure by interacting with the Flavin Mononucleotide site of complex I^64^. Moreover, work with murine models of compromised complex I activity has revealed that it plays a critical role in osteoclastogenesis. Indeed, Jin et al. examined the function of complex I in osteoclasts using mice lacking NADH: ubiquinone oxidoreductase iron-sulfur protein 4 (Ndufs4) in the myeloid lineage^65^. Ndufs4 is a nuclear-encoded protein critical for mitochondrial complex I assembly^66,67^. Deletion of Ndufs4 impaired osteoclast differentiation and caused high bone mass. This earlier work along with our present findings suggest that the anti-osteoclastogenic effect of estrogens is the result of reduced complex I activity. Future studies are needed to elucidate both the molecular mechanisms via which RANKL rapidly increases OXPHOS activity and the antagonistic effects of E_2_.

In contrast to their effects on osteoclasts, estrogens promote mitochondrial biogenesis and function in various other cell types from tissues such as the brain, heart, skeletal muscle and pancreas. In the brain and heart, estrogens enhance the efficiency of mitochondrial electron transport chain and attenuate mitochondrial ATP depletion and reduced membrane potential^68,69^ by modulating the expression of nuclear-encoded mitochondrial proteins or by activating cytoplasmic signaling pathways^70^. In both skeletal muscle and β-cells, ERα signaling promotes mitochondrial fission–fusion dynamics^71,72^. ERs associate with mitochondria in various cell types^73,74^. However, whether estrogens regulate mitochondria activity via mitochondria-associated ERα remains unclear.

We also show herein that rotenone, like estrogens, causes apoptosis of osteoclast progenitors via a Bak and Bax-mediated mechanism. The pro-apoptotic effects of rotenone are in line with its inhibitory actions on osteoclastogenesis and lipopolysaccharide-induced bone erosion^75,76^. Additional support for a critical role of compromised complex I activity on apoptosis is provided by the findings that macrophages and mature osteoclasts lacking Ndufs4 exhibited increased apoptosis^65^. Furthermore, deletion of the mitochondrial transcription factor A (Tfam) in osteoclasts reduces intracellular ATP levels and accelerates osteoclasts apoptosis in mice^77^. Importantly, the inhibitory effect of rotenone on osteoclast generation in the present work was greatly attenuated in cells lacking Bak and Bax, implicating apoptosis as a major consequence of complex I inhibition. The fact that rotenone mimics the effects of E_2_ on Bak/Bax dependent apoptosis, further supports the contention that inhibition of complex I is a critical mediator of the pro-apoptotic effects of estrogens on osteoclast progenitors. Be that as it may, we cannot rule out the contribution of other death pathways or alterations in cell cycle. Further studies will be required to examine the contribution, or lack thereof, of such alternative mechanisms to the anti-osteoclastogenic effects of estrogens.

Earlier studies of ours have shown that an E_2_ dendrimer conjugate (EDC), incapable of stimulating nuclear-initiated actions of ERα could not prevent the increase in trabecular bone resorption and the loss of trabecular bone following OVX^48^. These findings, along with the present results, suggest that nuclear actions of ERα mediate the effects of estrogens on the mitochondria of osteoclasts. Mitochondria complex I is a major source of reactive oxygen species (ROS)^78^ that, in turn, play a crucial role in osteoclast differentiation and function^79–83^. We have elucidated earlier that attenuation of mitochondrial H_2_O_2_ in osteoclast lineage cells contributes to the anti-resorptive and bone protective actions of estrogens^33,84^. The inhibitory effects of E2 on complex I activity provides a potential mechanism for the previously described actions of sex steroids in suppressing mitochondrial H_2_O_2_.

In conclusion, the work described herein suggests that the pro-apoptotic and anti-osteoclastogenic actions of estrogens result from the inhibition of mitochondria complex I activity in osteoclast progenitors. Future in vivo studies will be required to establish the contribution of complex I and osteoclast progenitor apoptosis to the bone protective actions of estrogens.

## Supplementary information

Supplementary file1

Supplementary file2

Supplementary file3

## Data Availability

The microarray data of this study is deposited at GEO database under Accession Number GSE111237 (https://www.ncbi.nlm.nih.gov/geo/query/acc.cgi?acc=GSE111237). Other datasets generated during the current study are available from the corresponding author on reasonable request.
