# Supplementary material for: Estrogens decrease osteoclast number by attenuating mitochondria oxidative phosphorylation and ATP production in early osteoclast precursors
Source: Sci Rep. 2020 Jul 20;10:11933. doi: 10.1038/s41598-020-68890-7 (PMC7371870; doi:10.1038/s41598-020-68890-7)
Supplement: Supplementary file 1 — Supplementary file1 [file 41598_2020_68890_MOESM1_ESM.pdf]

Supplementary data

Estrogens decrease osteoclast number by attenuating mitochondria oxidative phosphorylation and ATP production in early osteoclast precursors

Ha-Neui Kim[1], Filipa Ponte[1], Intawat Nookaew[2], Serra Ucer Ozgurel[1], Adriana Marques-Carvalho[3], Srividhya Iyer[4], Aaron Warren[1], Nukhet Aykin-Burns[5], Kimberly Krager[5], Vilma A Sardao[3], Li Han[1], Rafael de Cabo[7], Haibo Zhao[1], Robert L. Jilka[1], Stavros C. Manolagas[1,4,6,8], Maria Almeida[1,4,6,8]

[1]Division of Endocrinology and Metabolism, Center for Osteoporosis and Metabolic Bone Diseases, University of Arkansas for Medical Sciences, Little Rock, USA.

[2]Department of Biomedical Informatics, University of Arkansas for Medical Sciences, Little Rock, USA.

[3]CNC - Center for Neuroscience and Cell Biology, University of Coimbra, UC-Biotech, Biocant Park, Cantanhede, Portugal.

[4]Department of Orthopedic Surgery, University of Arkansas for Medical Sciences, Little Rock, USA.

[5]Department of Pharmaceutical Sciences, Division of Radiation Health, University of Arkansas for Medical Sciences, Little Rock, USA.

[6]Central Arkansas Veterans Healthcare System, Little Rock, AR 72205.

[7]Translational Gerontology Branch, NIA, NIH, Baltimore, MD, USA.

[8]Contributed equally.

Corresponding Author: Maria Almeida, Ph.D., University of Arkansas for Medical Sciences, Division of Endocrinology and Metabolism, 4301 W. Markham St. #587, Little Rock, 72205-7199.

E-Mail: schullermaria@uams.edu; Phone: 501-686-7856; Fax: 501-686-8148
