# Supplementary material for: Estrogens decrease osteoclast number by attenuating mitochondria oxidative phosphorylation and ATP production in early osteoclast precursors
Source: Sci Rep. 2020 Jul 20;10:11933. doi: 10.1038/s41598-020-68890-7 (PMC7371870; doi:10.1038/s41598-020-68890-7)
Supplement: Supplementary file 2 — Supplementary file2 [file 41598_2020_68890_MOESM2_ESM.pdf]

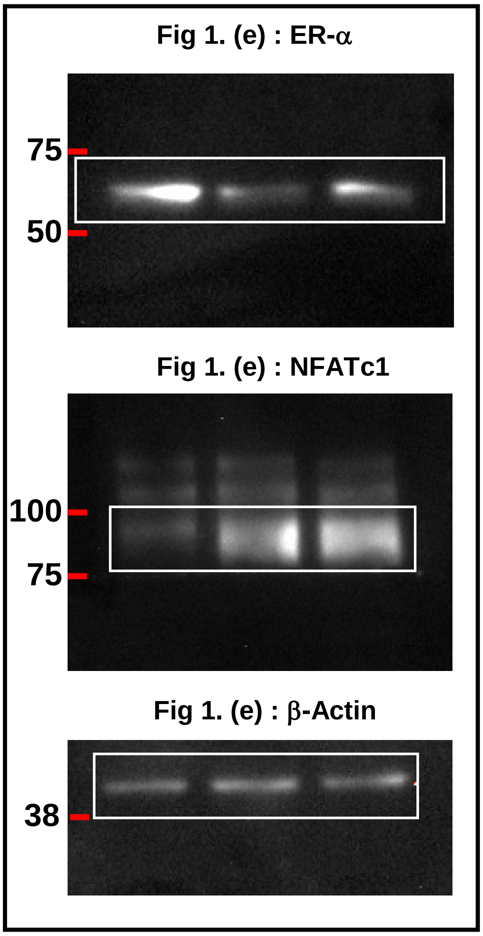

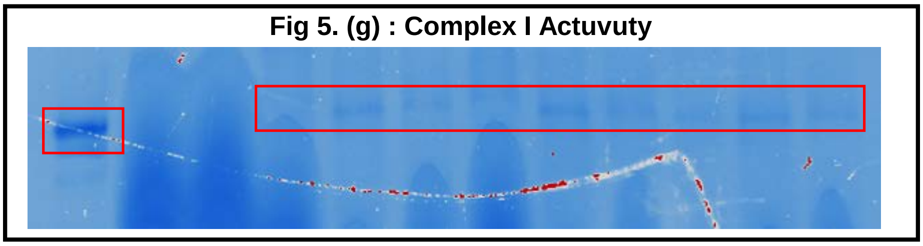

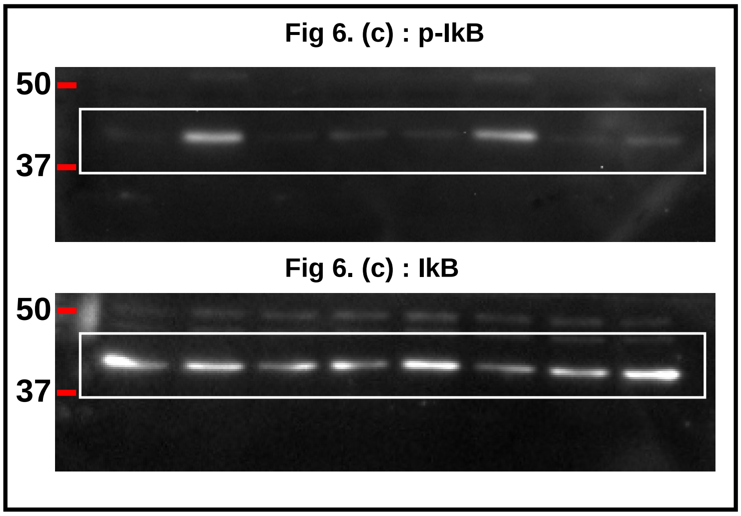

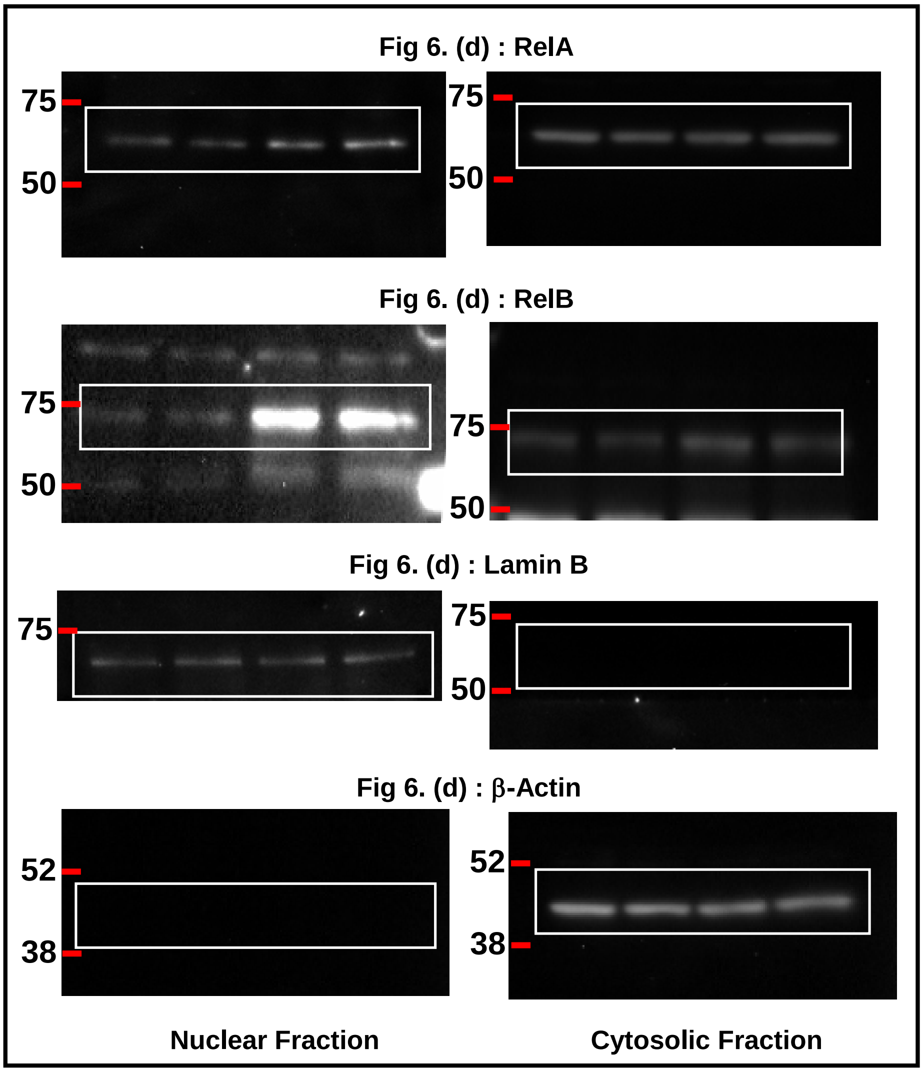

Supplementary Figure
