# Supplementary material for: Estrogens decrease osteoclast number by attenuating mitochondria oxidative phosphorylation and ATP production in early osteoclast precursors
Source: Sci Rep. 2020 Jul 20;10:11933. doi: 10.1038/s41598-020-68890-7 (PMC7371870; doi:10.1038/s41598-020-68890-7)
Supplement: Supplementary file 3 — Supplementary file3 [file 41598_2020_68890_MOESM3_ESM.pdf]

Supplementary Figure. ***The entire blots including molecular weight markers.*** Uncropped raw data for immunoblotting analyses shown in main Figures are provided. Red or white rectangles indicate the proteins which are shown in the main Figures. Each lane is represented in the legends. The Western blot presented is representative of more than two independent experiments.
